# Repertoires of Vaccine Refusal in Romania

**DOI:** 10.3390/vaccines8040757

**Published:** 2020-12-13

**Authors:** Cosmin Toth

**Affiliations:** Faculty of Sociology and Social Work, University of Bucharest, 030018 Bucharest, Romania; cosmin.toth@sas.unibuc.ro

**Keywords:** refusal of vaccination, interpretive repertoires, discourse analysis

## Abstract

Repertoires are basic analytic units in discourse analysis and discursive psychology, characterized as repeatable building blocks speakers use for constructing versions of actions. In this study my aim is to analyze public repertoires which are available to parents as discursive resources to substantiate their decision not to vaccinate their children. Online content, two televised talk shows and a series of interviews with parents who refused vaccination from 2017–2019 were analyzed. As a result of this analysis, I have identified a series of repertoires such as distrust repertoires, rejecting any risks when it comes to children, vaccine ineffectiveness and ‘immunity is a limited resource that should not be forced’. These repertoires do a discursive work that seem to go beyond signs of concern or challenges to vaccine safety to perform a moral and epistemic delegitimization of the current system of medical services, medical research and government authorities. Moreover, the identification of the repertoires that circulate in the public space as resources fulfil a discursive function of replacing the current system with new moral and epistemic perspectives.

## 1. Introduction

Since 2016, an increase in the number of measles cases in Romania has led the Romanian Ministry of Health to declare a measles epidemic. In the previous year, Romania had reached a historic minimum level of Measles-Mumps-Rubella (MMR) vaccine coverage. The 2019 National Institute of Public Health in Romania (NIPHR) report indicated a declining trend in MMR vaccine coverage from 95% in 2005 to a minimum of 86% in 2015., recovering to almost 90% in 2018 [1].

According to the latest NIPHR report (July 2020) [2], the number of measles cases has increased to 20,204 since the beginning of the epidemic. One possible cause that has been much debated is the explicit refusal of vaccination. In July 2019, explicit refusal of vaccination was responsible for 11.8% of the total number of children unvaccinated at 24 months [3]. A national poll conducted between 21 January and 11 February 2019 (INSCOP Research in Romania, commissioned by the National Society of Family Medicine [4]) found that only 55% of respondents believed that the benefits of vaccination outweigh the risks, while 8% believed that vaccination of children is not necessary.

This diversity of opinion has been reflected in public debates, either in broadcast media or across the internet, between representatives of the antivaccine movement, those hesitant about vaccination and those who have become advocates for vaccines. The major topic of discussion has been grounds for refusing vaccination, which is of interest to decision-makers, government officials, members of the public and any researcher who is concerned with the attitudes and actions of the population in the field of public health. Numerous researchers have tried to understand the reasons for hesitation and refusal to vaccinate children. Diaz Crescitelli et al. synthesized findings from 27 qualitative studies and found a number of opinions, including concerns about possible toxicity of vaccines, ideas that a healthy lifestyle renders vaccines useless, beliefs that preventable diseases are not particularly dangerous, distrust of authorities and a philosophy of life which orientates towards alternative forms of medicine [5]. Another synthesis showed that the main cause for concern is the side effects of vaccines [6]. Dubé et al. found that mothers who are hesitant about vaccination considered them to be ineffective and dangerous, and distrust medical authorities [7]. A meta-analysis of 145 articles which highlighted that the main concerns are related to vaccine safety in association with distrust of government authorities [8]. In Romania, the refusal of the free human papillomavirus (HPV) vaccine was driven by fear of being part of an experimental drug testing program [9].

Online debates provide not only a glimpse of the reasons for refusing vaccination, but also an important source of documentation to guide vaccination decisions. Thus, the vocabularies of motives [10] that circulate and prove to be acceptable and ultimately legitimate become a discursive capital available to people when making decisions about vaccination. Kata (2012) showed that as a result of the development of Web 2.0, associated with the postmodern perspective of the informed citizen, the influence of vaccination campaigns on vaccines has significantly increased [11]. Betsch et al. (2010) demonstrated experimentally that there is a significant influence of exposure to antivaccine messages on web pages about the perception of risks regarding vaccination [12]. Witteman and Zikmund-Fisher (2012) also showed how exposures to narratives can affect perceptions of vaccination risks [13].

The impact of the information available in the media and across the internet does not just simply occur via unreflective assimilation, but is also influenced by the manner in which it is constructed and articulated in legitimate discourses. Based on credibility and morality, these discourses eventually may become available resources for decision-makers. The present study therefore does not focus on attitudes, but on discourses and repertoires; they are considered to be much more than just channels of communication of opinions and attitudes, as they have a significant and essential formative character. This approach is consistent with that of discourse analysis (DA) and especially with discursive psychology (DP).

The DA perspective recovers the constructive capacity of language [14,15], challenging reductionist approaches which hold that language is a simple channel for communicating mental processes [15,16]. Attitudes and interests do not precede language, but emerge from discursive configurations and must be mobilized and made discursively available [17]. Thus, DA is the study of language-in-use, or more precisely, how language is used to do things [18,19], because discourse is the main medium of action [20]. The way in which language is used—the choice of vocabulary, grammar, syntactic structure, style figures, rhetorical devices and the type of narrative construction—produces consequences, sometimes unintentionally. Through language, explanations are given, justifications are offered, categories and members of these categories are created [21] and power is legitimized [22]. DA is an analysis of the functions of language. The aim of this research is to provide an insight into the main interpretative repertoires circulated and used in Romania for supporting child vaccination rejection.

## 2. Materials and Methods

Five parents were interviewed, based on recommendations from the author’s social network, especially due to the difficulties in finding parents with children up to 10-years-old willing to talk only about the decision not to vaccinate. The interviewed parents were all female, with higher education, from urban areas, with at least one child that was not vaccinated. The author conducted in-depth interviews, inviting the interviewees to express their opinion on vaccines/vaccinations and on how they reached the decision not to vaccinate their child/children. The interviewer introduced himself as a researcher who is interested in people’s opinions about vaccines and vaccinations. The interviewees were aware that they had been invited based on their decision not to vaccinate their child/children. The author made sure that the ethical norms for in-depth interviews were respected. Thus, interviews were confidential, with explicit informed consent obtained from the interviewees. The interviewer also took care to protect the respondents emotionally during the interviews, as well as adopting a neutral, nonjudgmental attitude towards the topics discussed. During the interview, the interviewer refrained from reactions or normative comments, using only minimal prompts (such as ‘can you describe that to us?’, ‘can you tell us how it happened?’, ‘can you give more details?’ or ‘how so?’) to encourage elaboration.

From the perspective of DP, the main unit of analysis is represented by interpretive repertoires. Repertoires were introduced as a conceptual and analytical unit by Gilbert and Mulkay (1984) [23] and were then taken over and developed by the promoters of DP. Repertoires represent building blocks speakers use for constructing versions of actions, cognitive processes and other phenomena [22], similar to books on the shelves of a public library, metaphorically speaking [15]. They represent linguistic resources in the form of a system of specific terms used in a specific stylistic and grammatical fashion [22]. They have a culturally familiar character [17], representing a basis for social understanding [15]. Another important feature is the recurrent nature or the habitual and familiar character [14,17], constituting linguistic resources that can be absorbed, circulated, refined and relaunched in the public space.

However, given that repertoires are ready-made linguistic corpora, they may contain information, arguments, metaphors and narratives that are readily taken and delivered in various persuasive contexts only because they are familiar and have proven their functionality in other contexts. The use of repertoires can become shortcuts with significant consequences for actions and decisions. Thus, repertoires build or can become part of discursively constructed social identities. These identities in turn prescribe lines of action. Repertoires can be used to legitimize certain positions or ideologies. They represent recurring vocabularies of reasons and motivations, of urging to action and of decision and of postfactum rationalization of decisions. Given the multiple consequences of repertoires circulating through multiple channels, their investigation is an important component for understanding how the antivaccine movement is constituted and the elements that are subsequently adopted by parents when making a decision about vaccinating their children.

In order to identify the repertoires as the main analytic tool of DP, I analyzed the written contents identified in the online environment, the transcription of the interviews and dialogues from the television shows. The analysis began with careful and repeated readings of the transcripts, searching for patterns and recurring organizations of discourse. Although we cannot speak in this case of rules and recipes [22], I followed discursive sequences composed of a restricted range of terms used in a specific stylistic and grammatical fashion, organized around recurring themes [22]. When people talk about a certain topic the same way, having the same lines of arguments, similar tropes and rhetoric devices indicate the existence of possible repertoires.

In order to capture the configuration of the repertoires for the present research, several stages were completed. The first phase started in 2017 with an analysis of online content available when searching for information based on several key terms generally associated with vaccines and vaccination, especially of children. This procedure was repeated in 2018, modifying some of the key terms. A third stage consisted of the analysis of two television shows, one from 2018 and one from 2019, which both invited a high-profile Romanian known for her critical position on vaccination. One of these shows focused only on reasons for hesitation regarding vaccination, while the second was more confrontational, as a doctor known for promoting vaccination was also invited. The last stage, in 2019, consisted of conducting interviews with five parents who had decided not to vaccinate their children.

I will further present the results of the analysis of the discourses obtained in each phase, identifying the repertoires in each discursive corpus. First, I analyzed the online content from 2017 and 2018, second, the transcripts of the two TV shows from 2018 and 2019, and finally, the transcripts of the interviews with the five parents.

## 3. Results

### 3.1. Online Content Analysis 2017 (Online Repertoires 2017—OR2017)

In June 2017, 52 internet pages were analyzed. Five antivaccine pages out of these were found. The antivaccine sites depicted vaccination using vivid metaphors and powerful rhetorical images (biological weapons, cellular poison, cancerous, neurotoxin, genetically-altered micro-organisms grown on pure mosquitoes). Vaccination was also described as being risky, offering no real protection, inhibiting the immune system; claims were made that unvaccinated children are actually healthier. Compared to the provaccine sites, the antivaccine sites offered more references, names and professional titles to support their scientific claims. Child-related discursive imagery was used, depicting vaccines as chemical substances that alter/affect fragile children in huge numbers so that they may suffer brain damage and die. Antivaccine sites accused officials of a lack of information, indifference, malevolence and of pursuing personal interests detrimental to public interests. Due to the volatility of such information, it was insufficient to outline a well-founded image of vaccine and vaccination discourses, and therefore not important enough to capture in detail. However, this analysis was performed rigorously, representing a starting point in the formation of a primary image and categories of repertoires.

In summary, the four categories of repertoires were (i) **vaccines are harmful**—metaphors and analogies with a strong negative rhetorical impact, such as cancer and toxins, pitted against the background of an oversized volume of scientific references; (ii) **vaccines’ ineffectiveness**—they do not do what they claim to do and unvaccinated children are actually healthier; (iii) **children with fragile bodies** are assaulted by vaccines; (iv) **distrust**—under the guise of accusations brought against Romanian governmental authorities.

### 3.2. Online Content Analysis 2018 (Online Repertoires 2018—OR2018)

A similar procedure to online content analysis was applied one year later. The amount of antivaccine pages retrieved via Google was similar to that of 2017. However, the antivaccine discourses seemed to use fewer metaphors and discursive imagery (bad for allergies, pollution, patent fraud). Vaccines continued to be described as being dangerous, risky and useless. However, what stood out were the magnitude and scale of the negative consequences associated with vaccination, through phrases such as ‘record number of…, ‘thousands of parents that…, ‘more cases of…, ‘overestimated benefits’, ‘significant losses’, ‘financial and human costs’, ‘important biological risks’, ‘continuous adverse effects’ and ‘disastrous effects’. There was also an abundance of statistics and data on various negative consequences of vaccinations and numerous mentions of conditions, diseases and side effects.

The two dominant repertoires can be summarized as (i) **alarmist but properly documented**—lots of empirical and research data, references and stories in a mostly formal journalistic language advising people to be aware that vaccines have serious negative effects at a large scale; (ii) **investigative-distrust**—stories about authorities, conflicting interests, conspiracies and financial interests. Compared to the previous year, there was a greater narrative dimension, with emphasis on amplifying the magnitude of the risks and the lack of credibility of both administrative and medical authorities.

### 3.3. First TV Show (TSR—Talk Show Repertoires)

On 17th March 2018, a prominent public figure (OS) was invited onto a television show to give a short speech (about five minutes) on vaccines. On 17th March 2018, a TV show was broadcast starting at 1 pm. The format of the show involved an interaction between a moderator who invited ‘OS’, a prominent public figure to talk about several topics (nutrition, raising children, etc.). During this show, OS was prompted to express her opinion about vaccines. The intervention lasted about 5 min and had the structure of a monologue. Currently the recording is also available on the internet with approximately forty-thousand views. OS is known for adopting a critical stance toward vaccines. In this short intervention, the main repertoire was that of the harmfulness of vaccines, especially associated with images of fatal diseases.

Analysis shows that OS employed the ‘vaccines are harmful’ repertoire, with discursive construction centered on the association of vaccines with very serious diseases, thus targeting a strong emotional effect:

I have nothing against vaccination. Today’s vaccines have nothing in common with the vaccines we were vaccinated with, immunized in Ceausescu’s time that came from the Cantacuzino Institute. In the vaccines of that time, if you had the opportunity to ask a pediatrician, maybe your next guest, he will tell you what the vaccines contained then, a doctor who caught both eras. What did the vaccines contain then and what does it contain today? How many children at that time had cancers and leukemia and how many have cancers and leukemia today? How harmful were the vaccines or what were the risks?

The discourse begins with a discursive position of moderation, supporting vaccination in principle, but rejecting today’s vaccines as being of inferior quality to those produced in Romania before 1989. The contrast is amplified by rhetorical questions to emphasize what should be considered obvious and a series of repetitions to create strongly negative discursive imagery of diseases ‘cancers and leukemia’. This builds the image of vaccines as harmful together with the apparent obviousness of this statement. This type of discursive construction has similarities with the repertoires identified in the online content from 2017, namely the repertoire of harmful vaccines counting on a strong emotional impact, particularly correlated with children depicted as victims.

A similar repertoire of vaccine harmfulness is constructed in the next fragment, which is also supported by an amplifier of credibility by invoking external authorities, considered a higher standard of seriousness than Romanian authorities. This is an example of the repertoire of moral and epistemic higher ground of foreign authorities, that recognized vaccine harmfulness.

We are talking…there are such cases it is true…we are talking about young girls aged 14,15,16 who had been injected with the vaccine against cervical cancer, young girls who were athletes, who were cheerleaders, were doing sports on daily basis, they were in perfect health and died from this vaccine. We must also say that in America there is a special court that judges such cases from its inception and until today compensation has been granted to vaccine victims, whether they were serious injuries or even deaths worth $4 billion. Do you know how such compensations are given? With evidence!

In this short intervention on the issue of vaccines, OS quickly creates an image of vaccines as harmful and demonstrates the validity of this statement by invoking a standard of American society which is portrayed as indisputable. The image of harmfulness is also accentuated by stylistic and rhetorical devices, such as rhetorical questions and repetition.

### 3.4. Second TV Show (TSR—Talk Show Repertoires)

On 21st March 2019, there was a televised debate between OS and a pediatrician known to the public not only for supporting vaccination but also for actively contesting antivaccine voices. The show was broadcast starting at 10.30, on a television channel among the top 3 in Romania. The format of the show involved a dialogue for and against vaccination moderated by the show’s presenter. The dialogue lasted about 30 min.

Although the debate was a dialogue, this analysis focuses on the main themes and repertoires activated by OS in the discussion against vaccines. As the topic is divisive and generates a strong dynamic of conversation, with many interruptions and forms of rhetorical aggression, the arguments and repertoires were not fully developed. Instead, they were mentioned in a condensed form and fragmented by interruptions.

OS begins with a repertoire of moderation and reasonableness through which she positions herself as a mother who has a series of questions. This identity allows her to challenge vaccines from a legitimate position—the generic, responsible parent—and thus it cannot be rhetorically challenged that she questions the vaccines on the basis of an ideological agenda or from academic-medical positions. Stating the intentions of a nonconflicting debate is an important ingredient of a balanced and objective position:

We are fasting for Easter. That is in the first place. So, I really want a calm discussion and in no case from frond positions or…pros and cons…uh…I would like to place myself today in the position of a mother who has some doubts.

In this context, OS introduces the idea that the sources of information are not the internet or some friend’s opinion, but the personal experiences of friends, together with a book on the subject that she presents in detail, repeatedly emphasizing its scientific legitimacy. This is an example of the properly documented repertoire:

Vaccines and Autoimmunity…a book written by medical researchers…Yehuda Shoenfeld is…global, his reputation is global, an undisputed immunologist and…I don’t know if he can be challenged or is questionable…however…who raises some very pertinent question marks and then, if you are a responsible parent.

The properly documented repertoire is also supported by invoking and collating scientific sources, in this case, drawing on an older theme of the relationship between autism and vaccination mediated by the presence of heavy metals. At the moderator’s challenge, the guest doctor denies the relationship between autism and the vaccine, challenging Andrew Wakefield’s study. OS intervenes in the discussion by invoking the fact that in the package leaflet of some vaccines, some side-effects relating to autism spectrum disorder are recognized. She performs a wide discursive work of legitimizing her own position by delegitimizing the sources and arguments of the interlocutor (the invited doctor):

There is no dilemma…look what he says…we can challenge any scientific researcher and based on this conclusion we can challenge the doctor here, we enter a dangerous carousel for anyone who has an opinion, yes? [What does this researcher say?]…He says so…it’s about…First of all, it starts from the statement of William Gies who 100 years ago came to the conclusion [yes] through personal observations, scientific studies that the use of aluminium or any aluminium compound in food is a dangerous practice…[quoting from the book)…the toxicity of aluminium discussed here is usually manifested by learning disabilities, memory, concentration and of speech, so problems that autistic children and those who have diseases of this spectrum, through diseases of the motor system, intensification of seizures and behavioural changes and so on.

In the passage above, OS begins by emphasizing the validity of the arguments, placing them at the same epistemic level with the doctor’s statements, through a discursive device of relativization. Then she continues the persuasive work by invoking the age of the source (100 years ago) as a significant feature. The essence of this discursive intervention is that of finally positioning the quoted source, through the frequent underlining of its quality, as superior to the doctor’s argument.

Towards the end of the discussion, OS also brings up a story about a friend who observed behavioral changes, which suggest some autism-related characteristics immediately after the vaccine was administered.

Just a second. I just have to tell you that my best friend has an autistic child, right? After the vaccine. The day before he had a favourite game on the table, coloured cards were placed and the child identified the horse and so on, and the child said horse, horse, horse because he was ordered to identify the horse. The next day after the vaccine he didn’t know what the horse was.

In this passage, through the first two introductory sentences, she announces the presentation of a crucial and defining argument. Succinctly, OS constructs an argument for the appearance of autism, legitimized this time by personal experience, presented as indisputable and irrefutable information, accentuated by a rhetorical question of confirmation. Invoking personal narratives such as ‘I know someone…’ creates a powerful discursive imagery that is emotionally impregnated and indisputable, because denying it would mean rejecting the honesty of the narrator, which is generally an unacceptable insult.

When the doctor mentions cohort studies of children on the effects of vaccination, OS immediately questions the validity of the results, asking rhetorically for the funding sources of these studies. The manifestation of immediate distrust toward these studies, by suggesting financial interests that could be in conflict of interest with the objective of the study, indicates a lack of confidence in the discourse of scientific authority that is related to pharmaceutical companies. This is an example of the ‘distrust due to conflict of interest’ repertoire.

Another repertoire is that of ‘fragile children’. This type of repertoire is useful for arguing against vaccination because it allows the classification of any risks, both those officially recognized and those circulated across the internet, as inacceptable because they apply to children. Thus, the blanket disqualification of vaccines becomes legitimate. At the doctor’s argument that the amount of aluminum in vaccines is much lower than that in breast milk, OS simply rejects this argument as unsatisfactory. With regard to total risk rejection, it suggests that only dedicated studies showing that vaccines are completely safe would be acceptable. The reproach used by OS, ‘we play with children’s lives’, invokes a social norm that is difficult to reject and consequently forces compliance with recognition that risks associated with vaccination cannot be accepted:

Doctor, if there are no safety studies to show that these 4 µg are safe it means that we are playing with children’s lives. Do you have any knowledge of any one study that shows that this proposed vaccination algorithm, that mandatory vaccines in our country are safe to be administered to children? All at once?

OS also mentions a theory about the functioning of vaccines, suggesting that it is possible to become infected with the virus directly as a result of vaccination. This leads to a discussion about the distinction between wild and derived viruses, and is an example of the ‘vaccine can cause the disease it is supposed to prevent’ repertoire. As a rhetorical strategy, a scientific study is required to prove that this is not the case:

But the doctor would do well to point out that there are two types of viruses, the wild-type virus and the vaccine-derived virus. You specified, or is it known whether the virus that infected those children came from the vaccine or is wild? To find out if it is not those that are vaccinated that make the unvaccinated sick?

[The doctor discusses the existence of such studies on doctors who have been contaminated]

Let me tell you, we are not sure that those children were infected with the wild-type virus and not the Varilix virus…

This does not mean that we have the certainty that those children became ill with the wild virus. If no studies have been done…so please let us talk based on evidence, okay? On evidence and science.

The speech is aggressive and accusatory, starting by suggesting a lack of transparency of the doctor and finishing by questioning the objectivity and scientific approach. The discursive work is oriented towards dismantling legitimacy, not only of the argument previously supported by the doctor but also of an unscientific attitude. It is a reproach and an attitude correction. For this, a generic ‘us’ is built discursively in opposition to the doctor and his arguments, thus asserting a majority superiority. Seen as a possible repertoire, this discourse suggests not only a theory about the functioning of vaccines but also distrust of doctors.

The main repertoires found in the analysis of television shows can be summarized as follows:

- Vaccines are harmful
- Moral and epistemic higher ground of foreign authorities that recognized vaccine harmfulness
- Moderation and reasonableness
- Properly documented
- Fragile children; victims of vaccines
- Vaccines can cause the disease they are supposed to prevent

### 3.5. Interviews with Parents (IR—Interview Repertoires)

This section analyzes the interviews with parents who decided not to vaccinate their children with any of the vaccines provided in the immunization scheme recommended by the Ministry of Health. It focuses on the most common repertoires, the way they are constituted and the discursive functions they fulfil. For the purpose of analysis, I have extracted from the transcribed discourses the fragments that seem to most correctly and synthetically exemplify the expressed repertoires.

One of the most widely used repertoires was related to the harmfulness of vaccines in terms of the risks they present. This is a repertoire of challenging and delegitimizing vaccines on the grounds of safety. There were two subtypes of repertoire, those that refer to the risks provided in the leaflet (mentioned by four out of five parents extensively) and those that refer to more or less uncertain medical consequences. Both are part of the main repertoire of ‘rejecting any risk when it comes to one’s own children’ (mentioned by all of the five parents interviewed).

In the example below, the discourse assesses the existence of any risk declared in the leaflet as unacceptable. Unacceptability is built by invoking one’s own child as the ultimate moral landmark. Within this repertoire, two categories of people are built: those who do not read the leaflet because they are gullible, naive and implicitly consider that vaccines are good, and those who read the leaflet and are amazed and scared by the stated side effects. The decision not to vaccinate is constructed as a process of ‘enlightenment’, of transition from a state of gullible ignorance to a state of astonished awareness. It constructs the idea that everyone who reads the prospectus will come to the same conclusion:

Have you seen or read what is written, out of curiosity…what is written on the vaccine leaflet? Well, when you read about aspirin, if you take the leaflet, you get scared. There are some things. Aaa and I received the argument, well yes, but the manufacturer is forced to put there everything. And isn’t this a normal thing to do? Yes, he’s too panicked, I don’t think he’s panicked at all, the man says what he saw when he tested the vaccine on his study groups, that is, there is what exists. Why would I put all these risks in a child’s small body and why? Why would I do such a thing?

I don’t want my children to be in those 2% or in those rare cases because no one, no one guarantees you that it will be a…that he will not be in those cases, that is, no doctor will sign that the child will be ok after vaccination and as long as there is this small risk, I prefer my child to get the measles.

A rhetorical question is used at the beginning to emphasize the evidence that the side effects are frightening and must be taken seriously. It is then stated that although these risks are unlikely, they cannot be ignored when it comes to children. This argument claims the legitimacy of rejecting any risk by discursively invoking one’s own child, thus building a moral and emotional image that is difficult to reject.

The other repertoire includes the rejection of risks of vaccination by reporting either known diseases such as autism, allergies, paralysis or other unknown effects. Autism is mentioned in one form or another in all discourses, along with heavy metals, but allergies are mentioned less. Controversial substances and unknown effects are invoked. The example below adopts a worried tone, citing plausible situations and hypotheses that have fueled uncertainty about the possible effects of vaccination. Narratives, or statements of some doctors, are presented to support the hypothesis of the link between vaccination and various diseases or effects:

Why a substance, that I do not know exactly what it contains, this is also a problem, that it contains aluminium, which is stored…I do not know how…that accumulates in…and there are too many question marks that create anxiety.

This is a possible repertoire in which effects such as known diseases are not strongly asserted, but at the same time, the concern expressed against unknowns is maintained. The tone is reserved regarding the assertion of the direct link with known and widely transmitted diseases in online antivaccination discourses, thus further presenting a rational-problematizing position. However, the assertion of unknown and worrying effects is strongly emphasized and presented as being unheard of, decisive and very worrying. The repercussions of vaccination risks overlap at least partially with the previous analyzes of the harmfulness of vaccines.

Interviews also contained examples of the vaccines are ineffective (four out of five parents) repertoire. In this category, the most prevalent argument cites ineffectiveness in preventing childhood diseases and a lack of, or limited, immunity:

Measles, rubella or flu vaccines…means…certain strains that are injected and you can make some easier form, but you can still get the disease, and I said to myself if I can still get the disease, many being childhood diseases, aaa…than no vaccination.

What strikes me is that there are children with…I don’t recall…yes…rotaviruses, and those children who are vaccinated and still got the disease as if they did not get any vaccine…

No vaccination gives you immunity for life, only the disease gives you immunity for life.

The wording indicates a type of scientific demonstrative repertoire, without using rhetorical devices, constructed in the form of logical evidence. This type of argument is built under the umbrella of a set of substitute theories that deny the effectiveness and usefulness of vaccination. This time the vaccine-preventable diseases are built as mild diseases, specific to childhood (the child is no longer a fragile character).

Another set of theories are those that challenge the effectiveness of vaccination as a medical invention that has contributed to improving public health (mentioned by two out of five interviewees). The idea that vaccines are responsible for lowering rates of illness and death is rejected in favor of hygiene, or the fact that certain diseases have disappeared on their own. The arguments are formulated as superior, alternative theories from an epistemic point of view, which better explain the reality compared to established but somewhat outdated traditional beliefs:

I think that this comparison with vaccines, that diseases have disappeared due to vaccines, that this is not the case, that hygiene has improved, that we live in an age when hygiene is very important and thus no one takes into account the hygiene conditions led to eradication of so many diseases…not necessarily vaccines.

By these discursive constructions, the popular and common theories that support the historical importance of immunization through vaccination for public health are delegitimized as being overestimated. The rhetorical construction emphasizes the revealing character of the new theory ‘no one takes into account’. Although the argument is more in the form of a hypothetical construction, it still benefits from a scientific-type rhetorical construction.

Continuing the line of arguments aimed at rationalizing the decision, further repertoires that deny the risks of nonvaccination were identified, namely ‘dangerous diseases have disappeared’ and ‘childhood diseases are mild’ (all five parents interviewed). It is argued that in the end, diseases considered dangerous such as polio, have disappeared, while the remaining childhood diseases do not pose significant risks. This means that there is virtually no risk from not getting vaccinated. Two types of arguments were put forward: stories from personal experience and information obtained from doctors that dangerous diseases have disappeared:

My biggest fear is polio, my husband says…yes but how many cases have you seen, it’s eradicated, it’s a whole medicine now, we don’t live the conditions of decades ago

I don’t understand why she shouldn’t be exposed to childhood diseases. I know a case…my colleague, uh, both children got chickenpox, and her husband of almost 40 years old also got chickenpox because he didn’t have it as baby, and…very unpleasant, exactly, the children got through easily, the youngest one the easiest, the other child mmm harder, harder than the youngest, and the husband was…awful, he would not wish this to his worst enemy. And then why?

The risks associated with nonvaccination are constructed as unrealistic, either because there are medical arguments that the most worrying diseases have disappeared, or because childhood diseases should be experienced as early as possible as they are more severe if experienced later in life. The first passage reproduces a dialogue with the interviewee’s husband. By introducing another character with moral right and rational authority, the lack of risk is built on the background of high-performance medicine. The second passage also tells a story from personal life, indisputable from the perspective of honesty, which emphasizes comparatively how the form of chickenpox was more serious when contracted by older people. Finally, a rhetorical question is used to emphasize the evidence and logical simplicity of the argument.

Further examples of discursive resources used to indicate a rational position in relation to immunization are variations of the repertoire on how the immune system and vaccines work (all five parents expressed theories about how immunization work). This takes two theoretical forms. One theory is that the immune system is a limited resource which weakens through recursive uses, or has limited resources that are ultimately used by vaccines. The interviewees build legitimacy by invoking doctors as a source of theories, statements or indications:

One immunologist…I can’t remember his name, I’ll remember. He is Romanian and he said the following…he had an interview in ‘Adevărul’ a few years ago, that the immune system is the least known, how the immune system works, that is. Scientists know only such a superficial part, they do not know profusely how it works, all its mechanisms and he made a very interesting analogy with vaccines aaa…Imagine an elastic that you keep pulling, and what will happen in the end? It will get weaker. That is, if you give your immune system…you keep fooling him with these vaccines…and he is alerted, in real fights he will be weakened and…I liked this analogy made by a doctor.

In order to legitimize his position, the interviewee begins by specifying the source as an immunology specialist, then continues with the description of the context (*Adevărul*—a reputable Romanian publication), the presentation of the theory about the negative impact of vaccines on immunity by analogy, returning at the end to re-emphasize the epistemic quality of the source.

The other theory is that the immune system is stronger if the body actually gets sick. This theory refers to the importance of catching diseases that are preventable by vaccination for the development of the immune system:

I do not have a specialized language, when I read, I understand the idea, but I cannot repeat the same…that these childhood diseases also have their role in the system. After having chickenpox or…you become stronger in the face of other diseases. I also read this…besides the fact that it offers you immunity for life, the immune system strengthens, because it goes through a real fight.

Thus, the role of childhood diseases, as a benefit of nonvaccination, is developed as a scientific truth, using analogies, taken from ‘read’ sources.

After risks of vaccination, the second most important issue is discourses of mistrust. Here, vaccination is associated with distrust in the medical system (doctors), in medicine and in vaccine manufacturers as well as in administrative authorities.

The accusatory discourse related to the doctors and the Romanian medical system is mentioned in two cases quite extensively, referring mainly to the pressure from doctors to vaccinate, the lack of explanations, the abuse of authority and the lack of information. A discursive image of the doctor lacking in transparency is constructed, subject to errors, meant to maintain the suspicion of vaccines and vaccinations.

I don’t…so no one in the doctor’s office told us if there was any adverse reaction, we asked her and she said that there will be no fever or irritability, that is, she was not open to tell us…well…look this can happen or look at the leaflet…

But there are families who read clinical trials and if, before, I was a patient who, whatever, the doctor told me I would have followed to the letter, now there are so many situations when I received the wrong diagnosis, wrong medication and unfortunately people die from wrong medication.

The first excerpt mentions a short story from personal experience accusing the doctor of a lack of openness. In the second, the construction shows a transition from the naive character who submits to authority to the character who understands that doctors make a lot of mistakes with deadly consequences. Legitimacy is thus deconstructed on two axes, the lack of transparency and the lack of professionalism of doctors.

Distrust of pharmaceutical manufacturers is built on the suspicion that they are eliminating or discrediting research that is not aligned with their own perspective (mentioned by four out of five parents). These repertoires are built on the background of mentioning the possession of documented but unspecified information about cases in which various pieces of research are not considered or rejected. Although it does not specify how these conclusions were reached, they are framed as being obvious:

Yes, yes in the end I noticed that all doctors, scientists, who come with a different opinion, are a little, that is not a little, they are totally taken off the line, they are discredited, and then I cannot trust.

There are studies that prove…were banned, banned, there were also publications in scientific research, journals, medicine, I don’t know where in the USA it seems. and this is the thing that these studies that are done for vaccination and I don’t know now are divided opinions, but I don’t know if they are all made by independent companies, which have nothing to gain from this thing, and as we all know, the pharmaceutical industry is very profitable.

Here, it is indirectly indicated by personal observations that pharmaceutical companies encourage and fund only research that can bring them profit, discrediting those that could reduce their earnings, thus being driven by illegitimate interests. This argument is made in a hesitant tone, but it is suggested that the lack of trust is logically justified.

Distrust of the authorities is also mentioned in relation to a lack of transparency, nonalignment with European standards and poor quality of services (mentioned by four out of five parents). The reproaches are formulated in a resigned tone in which although there is a willingness to trust, this becomes impossible because of a lack of transparency or incompetence:

Doubtful source of vaccines and there may be a lot of fake news, but as long as the Ministry of Health is not able to come and say ‘Sir, this is the source, from there I took the vaccines, they were kept in these conditions, I distributed them like this’, and I no longer trust the Romanian state.

Let’s align ourselves with the European Union, let’s see, not in vain 20 countries have removed and banned the ‘Infanrix’ and use its alternative, yet we still do it.

The rhetorical construction is not aggressive, but rather reproachful, not being denied good faith, thus indicating a balanced position of accepting the possibility that the accusations are based on false news.

Almost all the parents interviewed present their position as moderate, reasonable and nonmilitant in terms of the use of allopathic medicine when needed [24]. These positions express a desire to be perceived as rational and responsible and can be interpreted as reactions to the accusations brought in the public space against antivaccinators as being irrational, uneducated and irresponsible. They are built as reactions of a minority:

When I see that he has a high fever, I’m not just standing there, I have a subscription, I go to the emergency room. If the doctor tells me that he needs to be supervised, I’m ready to hospitalize him. I do NOT give him homeopathic pills leaving him with fever of 40 °C. Of course, I am also with allopathic medicine, with antibiotics.

I personally do not recommend either to anyone, I only say why I have not vaccinated so far.

I’m not against vaccination, I really think it’s a very good thing, but I want a doctor to make sure it’s 100% safe.

Another repertoire, aiming to construct the identity of the rational and responsible parent is that of the proper documentation: ‘I go to the source, I don’t rely on the internet’ [24]. In this type of construction, parents make discursive efforts to demonstrate the quality and volume of sources they have investigated, positioning themselves in opposition to an artificially constructed category of ‘those who believe anything they read on the internet’. For authors of sources they have consulted, parents provide scientific titles and describe previous contributions and biographies, sometimes in a hyperbolic manner.

### 3.6. Identified Repertories and Their Functions

On the surface, the rhetorical constructions made by antivaccination arguments at the declarative level aim only to question the decisions of the official system and to express a series of concerns. However, the analysis presented here shows that the repertoires developed seem to go beyond this aim. They constitute constructions steered at legitimizing the decision not to vaccinate, which ultimately lead to epistemic and moral constructions aimed at dislocating and subsequently replacing the system of moral and scientific legitimation of official authorities with a system of their own.

The picture is therefore one of rhetorical efforts to dislocate, by discrediting both the moral capacity of the medical and governmental authorities to make decisions, and the rationality, i.e., scientific legitimacy, of their decisions. Some of the identified repertoires fulfil functions of denial of moral authority: repertoires of suspicion and distrust in the political system, in the medical system and in the scientific legitimacy of doctors. There are also repertoires that aim to emphasize the harmfulness of vaccination by building images with strong emotional impact, warning about dangers to society and to children in particular, which imply that there is an immorality associated with using such substances. Other repertoires primarily seek to challenge vaccines at an epistemic level, by questioning their effectiveness, denying the risks of nonvaccination and invoking institutions endowed with an indisputable moral and epistemic stature (for example, courts in the United States) that have used scientific evidence to demonstrate harmfulness.

Along with the constructions of moral and epistemic delegitimization, there is a series of repertoires designed to establish a new moral and epistemic authority, superior to that of the main formal authorities and institutions. From a moral point of view, these include repertoires of moderation and reasonableness, unbiased and balanced decisions, and repertoires emphasizing moral superiority by invoking the generic discursive child who cannot be exposed to any risk for fear that s/he will be a fragile victim of immunization policies. At the epistemic level, scientific theories are elaborated and described in repertoires proposing that immunity is a limited resource or that the unvaccinated gain stronger natural immunity and the repertoire that vaccines that can cause the disease they are supposed to prevent. The repertoire of a well-documented parent, capable of accessing scientific sources that are not taken from the unreliable internet, completes the epistemic picture. As shown previously [24], these repertoires allow parents to build an identity of the loving, responsible and rational parent, morally (and even rationally and scientifically) superior to governmental, medical and research authorities.

**Figure 1:**
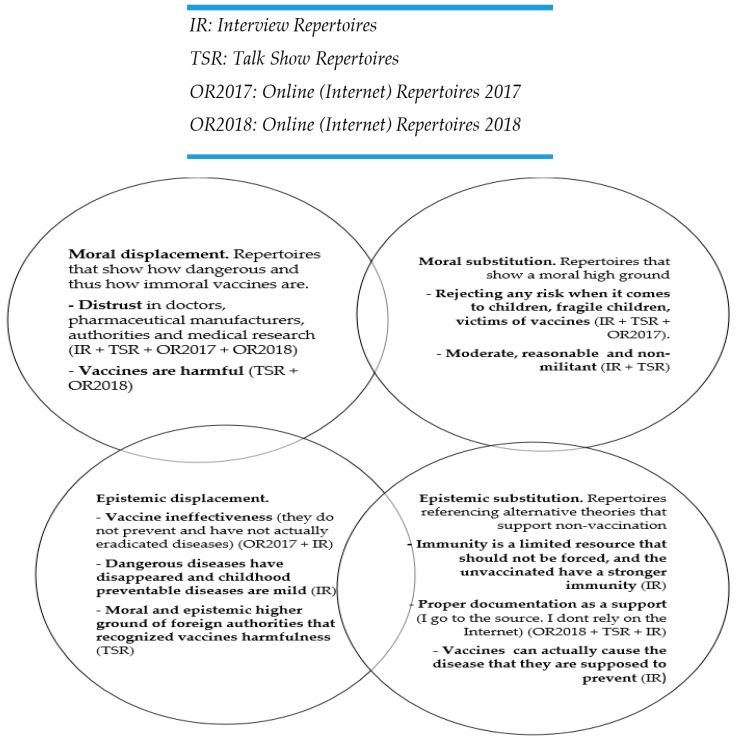
Identified antivaccine repertoires grouped according to discursive legitimizing-delegitimizing functions.

Although these repertoires are treated for analytical reasons as fulfilling distinct functions, they partially overlap and fulfil multiple functions simultaneously (Figure 1). In presenting the danger posed by vaccines, there is a moral and epistemic dislocation, although the epistemic dislocation is made to a lesser extent. The repertoires of moral and epistemic dislocation contribute to a subtle and gradual preparation for replacement with one’s own perspective and positioning. Similarly, the invocation of institutions such as American courts mainly serves to dismantle epistemic claims that support the safety of vaccines, but also fulfils the function of moral challenge by asserting the moral superiority of these types of courts. The repertoire regarding good documentation and lack of reliance upon the internet performs a moral legitimation (I do my duty to inform myself) and epistemic legitimation (I am well-informed from reliable sources).

Although these functions subtly intertwine, there is an identifiable thread of increasingly refined construction, that of a gradual substitution of the epistemic and moral legitimacy of government institutions and of the main scientific paradigms, with one of a “superior” lay person’s epistemic and moral system.

## 4. Discussion

Since the Romanian authorities’ declaration of a measles pandemic in 2016, public debates in Romania on vaccination have begun to intensify. This study investigated the discourses circulating in the country on the topic of child vaccination, especially among people who are hesitant or refuse vaccination. These discourses take the form of repertoires that in turn become resources for parents who are in a position to decide about immunizing their children. I identified several such repertoires and found that they not only fulfil local functions, i.e., justification, but also offer moral and epistemic alternatives to the mainstream discourse of medical and government authorities which support vaccination.

The repertoires identified were multiple, thus constituting a varied discursive resource available to parents. This is particularly notable since this topic may place parents in a vulnerable situation, sometimes confused from an informational perspective [25]. One of the main repertoires identified in this research was represented by the ‘distrust’ repertoires, in doctors, pharmaceutical manufacturers, authorities and medical research. The issue of distrust has been found by others to be one of the dominant attitudes of refusal or hesitation in the face of vaccination [5,7]. Similarly, other arguments and reasons, which resonates with the identified repertoires have been highlighted, such as fear of side effects [6,7] and the perception that children are too precious to subject to risk [26,27]. Confirming the existence of these attitudes among the Romanian public is undoubtedly important. Through the present study, however, I have offered an image of the rejecting discourses, brought under the same umbrella and presented in the form of a pool of easily accessible resources. Repertoires are not treated as reflections of attitudes, but serve as ready-made functional discursive constructions, which can be easily employed without reflective effort. I therefore suggest the need for a dramatic reconsideration of the importance of language in understanding the ways in which social reality is constructed, decisions are substantiated and actions are performed. Although there are some notable studies which have used a discursive approach to explore the topic of vaccination [28,29,30], it is still an underemployed methodological approach in this field and could bring new perspectives on how the foundations of decisions are built and actions are taken.

A second step of the study was represented by the classification of the identified repertoires according to the functions they perform. Some of the repertoires came to delegitimize existing official discourses morally and epistemically, while another category offered new possible alternatives. The repertoires do not only fulfil local contextualized functions, but also global functions of ideological displacement. A series of papers have shown that antivaccine attitudes and hesitation about vaccination can no longer be approached as simple cases of irrationality, simple possibility judgments [26], from positions of epistemic superiority or in sarcastic treatises [31,32]. Blume (2006) suggested that the refusal of vaccination should not only be seen as a result of the influence of the vaccine movement but rather as a consequence of a paradigm shift in public health ideology, according to which citizens have been encouraged to become responsible, informed, critical and empowered consumers [33]. Kata (2012) referred to a postmodern approach that emphasizes patients’ ability to inform themselves and to contribute to the knowledge base [11]. The effects of this are represented by the delegitimization of expert authority, replaced by expertise of ordinary people. The identified repertoires not only confirmed such an approach, but also indicated the concrete way in which such ideological change is articulated at a discursive level. In this context, for citizens who are invited to be critical, informed and challenging, repertoires represent ready-made content in open circulation, morally and epistemically grounded, which can be accessed as discursive resources that allow them to become ‘experts’.

## 5. The Limitations of the Study

This study has two main limitations. One is related to the specific methodology of discourse analysis that operates with mechanisms of interpreting the meaning and functions that various discursive sequences produce. These meanings are never fixed and are often open to contestation and negotiation. Therefore, this research cannot claim to have definitively and completely identified the meanings and functions of the analyzed repertoires. However, concentrating as much as possible only on the analysis of the text in front of me and on the function that it may fulfil, represents a methodological prescription of discourse analysis that limits interpretive excesses.

Second, discourse analysis focuses on the way in which discourse is constructed at the microlevel and is not intended to be statistically representative or generalizable. However, working with a larger volume of discursive sequences allows for the identification of regularities and patterns useful in understanding how discourses and language work. This is all the more important as in this study the repertoires were studied as discursive sequences characterized by recurrence. This study provides an image of the repertoires used in Romania for rejection of and hesitation around vaccination, however extending the research to include more sources will deepen our understanding of the phenomenon in question and strengthen conclusions.

## 6. Conclusions

The aim of this research was to identify the repertoires circulating in Romania of rejection of vaccines applied to children, as well as the functions they perform. Several repertoires were identified, some occurring within all the studied contexts (online content, TV shows and interviews). It seemed that these repertoires managed both to delegitimize a series of dominant discourses (of the main actors in the health systems) and to offer moral and epistemic alternatives. These findings have important implications. The identified repertoires are easily accessible discursive resources available to any parent faced with the decision to vaccinate their children. They are ready-made answers to challenge the benefits of vaccination, for anyone who is hesitant or just curious about vaccination. Understanding these repertoires can be an important starting point for main actors in the health system to adopt adapted discursive contents in the form of specific responses to each repertoire. Possible suggestions included accepting dialogue, demonstrating transparency, familiarizing the public with the results of medical research through accessible discourses, understanding alternative theories and challenging them by providing accessible versions rather than dismissive attitudes from positions of epistemic superiority [32]. This study is a starting point for future research into the importance of language for understanding health-related decisions and behaviors, especially in a world where the internet is a preferred source of information for many people.
